# Psychological stress induces depressive-like behavior associated with bone marrow-derived monocyte infiltration into the hippocampus independent of blood–brain barrier disruption

**DOI:** 10.1186/s12974-022-02569-w

**Published:** 2022-08-24

**Authors:** Huiling Hu, Xue Yang, Yuqing He, Chaohui Duan, Nannan Sun

**Affiliations:** 1 Department of Clinical Laboratory, Sun Yat-Sen Memorial Hospital, Sun Yat-Sen University, Guangzhou, China; 2 Guangdong Provincial Key Laboratory of Malignant Tumor Epigenetics and Gene Regulation, Sun Yat-Sen Memorial Hospital, Sun Yat-Sen University, Guangzhou, China; 3 State Key Laboratory of Ophthalmology, Zhongshan Ophthalmic Center, Sun Yat-Sen University, Guangzhou, China; 4 Translational Medicine Research Center, Zhujiang Hospital, Southern Medical University, Guangzhou, China; 5 Department of Obstetrics and Gynecology, Guangdong Provincial Key Laboratory of Major Obstetric Diseases, The Third Affiliated Hospital of Guangzhou Medical University, Guangzhou Medical University, Guangzhou, China

**Keywords:** Psychological stress, Depression, Bone marrow transplantation, Monocytes, Blood–brain barrier, Hippocampus

## Abstract

**Background:**

Psychological stress is one of the most important factors that trigger emotional disorders, such as depression and anxiety. Emerging evidence suggests that neuroinflammation exacerbated by bidirectional communication between the peripheral immune system and the central nervous system facilitates abnormal psychiatric symptoms. This study aimed to investigate the hippocampal migration of bone marrow (BM)-derived monocytes and its role in regulating depressive-like behaviors using the chronic psychological stress (CPS) mouse model. More importantly, whether the central migration of these peripheral BM-derived cells depend on the disruption of the blood–brain barrier (BBB) was also investigated.

**Methods and findings:**

Green fluorescent protein-positive (GFP^+^) BM chimeric mice were used to distinguish BM-derived monocytes within the brain. A CPS mouse model was established to explore the effect of CPS on hippocampal migration of BM-derived monocytes and its role in the regulation of depressive-like behaviors. The results revealed that BM-derived GFP^+^ cells accumulated in the hippocampus and differentiated into microglia-like cells after exposure to CPS. Interestingly, this migration was not associated with BBB disruption. Furthermore, treatment with C–C chemokine receptor 2 (CCR2) antagonist (RS102895) suppressed the recruitment of BM-derived monocytes to the hippocampus and alleviated depressive-like symptoms.

**Conclusion:**

These findings indicate that monocyte recruitment to the hippocampus in response to psychological stress may represent a novel cellular mechanism that contributes to the development of depression.

**Supplementary Information:**

The online version contains supplementary material available at 10.1186/s12974-022-02569-w.

## Introduction

Major depression is a multifactorial and heterogeneous mental disorder, characterized by episodes of depressed mood, anhedonia (loss of interest and pleasure), cognitive dysfunction, and a tendency to suicide [1]. Negative stimuli such as chronic physical and psychological stress are considered as important factors contributing to the development of this psychiatric disorder. A large body of post-mortem and neuroimaging studies of depressed patients have shown that various emotionally regulated brain regions are implicated in depression, including the prefrontal cortex (PFC), hippocampus, striatum, and amygdala [2, 3], of which the hippocampus has attracted significant attention for its role in declarative memory, spatial learning, and production of neurotrophic factors [4]. The hippocampus is densely populated with receptors for stress hormones, especially glucocorticoid receptors, and plays a vital role in the feedback regulation of hypothalamic–pituitary–adrenal (HPA) axis [5]. Under normal physiological conditions, HPA axis is activated in response to stress, causing secretion of glucocorticoids, which provide acute phase maximum physiological support for the fight or flight reaction. However, the excessive synthesis and release of glucocorticoids in response to repeated stressful experiences disrupts the negative feedback mechanisms of HPA axis, causing functional damage to the hippocampus [6]. Recent studies have shown that decreased hippocampal neurogenesis occurs in response to both acute and chronic stress, effects which appear to be mediated by glucocorticoids [7–9]. The memory impairments that occur in major depressive disorders appear to be the most intuitively obvious potential clinical symptom correlated with hippocampal neurogenesis, while the impairments of neurogenesis might also contribute to other clinical features of depression [10–12]. However, the precise role of the hippocampus in regulating depressive symptoms in response to chronic stress has not been comprehensively investigated.

Accumulating evidence has shown that microglia and bone marrow (BM)-derived monocytes play key roles in the behavioral function of diverse psychiatric disorders [13–15]. Studies using murine models of chronic stress induced by foot shock, social defeat stress, and chronic unpredictable stress indicated that microglia displayed activated morphology corresponding with increased expression of pro-inflammatory cytokines and exacerbated immune responses [13, 16–18]. Moreover, stressed microglia potentiate HPA axis activation, which together with the sympathetic nervous system (SNS) transfer stress signal to the peripheral immune system [19]. Chronic stress-induced persistent release of catecholamines can flux into immune organs, including the BM, where cells express its receptor. Once the interaction between ligands and receptors occurs, the production of myeloid cells is promoted [20]. Monocytes derived from BM then shift their phenotype to be more inflammatory and enhance their trafficking throughout the body [21]. Studies have revealed that the brain injury and infection as well as psychological stress trigger the recruitment of BM-derived monocytes into the brain and spinal cord [17, 22, 23]. At present, it is possible to define three distinct routes for leukocytes entry into the brain: (i) from blood to cerebrospinal fluid (CSF) across the choroid plexus; (ii) from blood to the subarachnoid space through meningeal vessels, and (iii) from blood to parenchymal perivascular spaces directly crossing the blood–brain barrier (BBB) [24]. Specifically, C–C chemokine receptor 2 (CCR2), which belongs to the family of G-protein-coupled, seven-transmembrane-spanning cell surface receptors, is required to mediate monocyte chemotaxis and plays an important role in inducing monocyte migration from the peripheral blood to the inflammatory sites through binding C–C chemokine ligands (CCL), among which CCL2 is the most potent for triggering signal transduction mediated by CCR2 [25, 26]. Besides haematopoietic cells, both CCR2 and CCL2 are also expressed on brain cells such as neurons, microglia and astrocytes, participating in the occurrence and development of a variety of neuropsychiatric disorders [27–29]. In Alzheimer’s disease, monocytes migrate from the bone marrow into the brain in a CCR2-dependent manner and contribute to accumulation of microglia [30]. Similarly, increased CCL2 expression in cerebrospinal fluid correlates with the severity of multiple sclerosis [29], and in experimental autoimmune encephalomyelitis animal model, knocking out CCR2 inhibits monocyte infiltration in the inflamed CNS [31]. CCR2A and CCR2B are two alternatively spliced forms of CCR2, the latter accounting for 90% [32]. RS102895, a potent and selective antagonist of CCR2 with high affinity to its β-subunit, has been widely used to interfere with CCR2 signaling in the brain [33]. Our previous study demonstrated that antibiotic-induced microbiome depletion disrupts the BBB and facilitates infiltration of BM-derived monocyte into the brain, whereas the concurrent treatment of RS102895 prevents CCR2-bearing monocyte trafficking into the brain [34]. However, whether the central migration of peripheral BM-derived cells entirely depends on BBB disruption remains unclear.

In this study, we found that BM-derived GFP^+^ cells accumulated in the hippocampus and differentiated into microglia-like cells after exposure to CPS. However, this migration was not associated with BBB disruption. Furthermore, treatment with CCR2 antagonist (RS102895) suppressed the recruitment of BM-derived monocytes to the hippocampus and alleviated depressive-like symptoms. These findings indicate that monocyte recruitment to the hippocampus in response to psychological stress may represent a novel cellular mechanism that contributes to the development of depression.

## Materials and methods

### Experiment animals

A total of 136 male C57BL/6J mice were used in this study across four separate cohorts (Additional file 1: Table S1). All mice were purchased from Beijing SPF Biotechnology Co., Ltd. (Beijing, China) at 5 weeks of age and allowed to adapt to their new environment for 1 week before the start of any experimental procedure. The mice were housed 4–5 per cage in specific pathogen-free conditions, maintained on a 12-h light–dark rhythm under controlled temperature (22–24 °C) and humidity (45–55%), with food and water available ad libitum and bedding replacement twice a week. Efforts were made to reduce the number of animals used and minimize animal suffering. All experimental procedures were approved by the Institutional Animal Care and Use Committee in the Zhongshan Ophthalmic Center of Sun Yat-sen University.

### Experimental design and treatment schedule

The central migration of BM-derived cells and BBB disruption induced by CPS was investigated in the first cohort containing 40 mice (cohort 1). Four weeks after BMT, these mice were randomly assigned to 2 groups: (1) the CPS group (*n* = 20 mice), treated with inescapable foot shocks for 5 consecutive days: (1) the control group (*n* = 20 mice), exposed to the same behavioral chamber in the absence of any foot shocks. In each of the two groups, 5 mice were used for quantification of recruited GFP^+^ cells in the hippocampus, 3 mice were used for observation of differential characteristics of infiltrated microglia-like cells, 4 mice were used for gene expression of enriched microvessels, 5 mice were used for occludin and ZO-1 immunostaining of isolated microvessels, 3 mice were used for measurement of BBB permeability with Evans blue dye. It should be noted that 4 mice among the control group were also randomly designated for chimerism evaluation within peripheral blood.

The inhibitory effects of CCR2 antagonist RS102895 on brain infiltration of BM-derived monocytes and depressive-like behaviors triggered by CPS were examined in the second and third cohorts (cohort 2 and 3). The two corresponding cohorts consisting of 76 mice (cohort 2 and 3) were divided into 3 groups at random 4 weeks after BMT: (1) the RS102895 group (*n* = 16 mice in cohort 2, *n* = 14 mice in cohort 3), treated with inescapable foot shocks for 5 consecutive days concurrent with RS102895 administration every 12 h starting from 24 h before CPS exposure and lasting for the following 6 days; (2) the CPS group (*n* = 16 mice in cohort 2, *n* = 14 mice in cohort 3), treated with inescapable foot shocks for 5 consecutive days concurrent with vehicle gavage; (3) the control group (*n* = 16 mice in cohort 2, *n* = 14 mice in cohort 3), treated with sham exposure concurrent with vehicle gavage. In each of the three groups, 4 mice from cohort 2 were used for quantification of infiltrated GFP^+^ cells in the hippocampus, 12 mice from cohort 2 underwent open field test (OFT), forced swim test (FST) and sucrose consumption test (SCT) successively for the assessment of depression phenotypes, 14 mice from cohort 3 were subjected to sucrose preference test (SPT) for a direct measurement of anhedonia.

The effects of RS102895 on depressive-like behaviors per se (without CPS exposure) were evaluated in the fourth cohort comprising 20 mice (cohort 4). Four weeks after BMT, they were split into 2 groups at random: (1) the RS102895 group (*n* = 10 mice), dosed twice daily for 15 consecutive days by oral gavage with 5 mg/kg of RS102895; (1) the vehicle group (*n* = 10 mice), orally treated with 100 μl of vehicle via gavage for a total of 30 doses. All mice in this cohort were used for behavioral testing of OFT, FST and SCT in sequence to check for the onset of any depressive symptom.

### Generation of GFP^+^ BM chimerism

High-level BM chimerism in mice was were performed as previously described [34, 35]. Briefly, to generate niche space within the recipient BM to allow for donor cell engraftment, BM cells of the wild-type C57BL/6J recipient mice (6 weeks old) were ablated by intraperitoneal injection of a freshly prepared busulfan dilution (30 mg/kg) once daily for 2 consecutive days. Two days after the second dose of busulfan conditioning, donor BM cells were harvested from tibias and femurs of adult male GFP transgenic mice (8 weeks old) and passed through 70-μm cell strainers. Subsequently, 150 μl of cell suspension containing 5 × 10^6^ GFP^+^ BM cells was gently injected into the lateral tail vein of conditioned recipient mice. Successful reconstitution of hematopoiesis was verified by flow-cytometric analysis and cytospin assay of GFP expression in peripheral blood leukocytes 4 weeks after BM transplantation.

For flow-cytometric analysis, 100 μl of blood was collected with heparin coated micro-centrifuge tube and then 1 ml PBS was added. Blood samples were centrifuged for 5 min at 400*g* and then the pellet was resuspended in 1 ml of erythrocyte lysing buffer. After 10 min, 1 ml of FACS buffer was added to quench the lysing buffer. The samples were centrifuged at 400*g* for 5 min and then were washed and resuspended in FACS buffer for analysis with BD AccuriC6 cytometer.

### Chronic psychological stress (CPS) exposure

Electric footshock stress, well-established psychological stress that induces behavioral deficits attributed to the state of learned helplessness, is a useful tool in neuropsychiatric studies [36]. To produce CPS, a chronic paradigm of electric foot shock was performed as previously described with some modifications [37]. Briefly, mice were subjected to inescapable footshock stress of varying intensity and duration on an electrified grid floor in a multi-conditioning chamber. After a 5-min adaptive phase, 360 intermittent, inescapable foot shocks with an intensity of 0.2 mA, variable durations of 1–5 s, and variable intervals of 1–15 s were delivered to mice in the CPS group in 60 min for 5 consecutive days. The control group mice were held in the same chamber for 65 min but received no stimulation. After foot shock or sham exposure, mice were subsequently returned to their home cages and left undisturbed. Before each mouse was introduced, a 75% ethanol solution was adopted to wipe the chamber to avoid any effects of feces and odor.

### Behavioral test

In accordance with the schedule described previously [38, 39], the behavioral tests were performed in the sequence of OFT, FST and SCT to minimize the potential carry-over effects.

### OFT

The OFT was performed to examine the exploratory activities and anxiety-like behavior in mice. After the acclimation period, the subject mouse was placed in the center of a square acrylic box (50 × 50 × 50 cm^3^) divided into 16 equal units. Each mouse was allowed to explore the box freely for 5 min, and its trajectory was automatically recorded by a video camera directed at the box. Between each trial, the apparatus was thoroughly cleaned with 75% ethanol to eliminate olfactory cues from the previous test. The total distance traveled by the mouse and the time spent in the central area (25% of the total area) were analyzed using an EthoVision video tracking system (Noldus).

### FST

The FST was used to evaluate despair or depressive behavior in mice. The mice were individually placed in a transparent plastic cylinder (15 cm diameter × 25 cm height) filled with fresh warm water (24 ± 1 °C) to the height of 25 cm and videotaped for 6 min. Immobility time during the last 4 min was quantified by two researchers unaware of treatment assignments. The immobility of mice was considered as floating in the water with no additional activity other than slightly moving to keep their head above the water. After the test, each mouse was dried thoroughly with a towel before being returned to its home cage.

### SCT

The SCT was conducted to assess the behavioral response related to anhedonia. On the first 2 days, each mouse was singly housed and habituated to 1% sucrose solution (w/v). After 12 h of inaccessibility to food and water, the mouse was immediately given the identical sugar water for 1 h in a quiet and peaceful environment. Sucrose solution consumption was quantified by weighing the drinking bottle before and after test period.

### Administration of CCR2 antagonist (RS102895)

A small molecular antagonist of CCR2 receptor, RS102895 was purchased from Sigma Aldrich. The dose and duration for RS102895 administration was decided upon previous reports proving considerable inhibitory effects on MCP-1/CCR2 interaction and abnormal chemotaxis in the brain triggered by sorts of physical and psychological stressors in rodents [40–44]. The 10 mg/ml stock solution was prepared in dimethyl sulfoxide (DMSO) as previously described [34, 44], and then diluted in sterile distilled water to final concentrations for use. The working solution of RS102895 (5 mg/kg in 100 μl) or an equal volume of vehicle was administrated to recipient mouse via gavage every 12 h starting from 24 h before CPS exposure and lasting for the following 5 days.

### Isolation of microvessels from the hippocampus

Microvessels from the hippocampus were isolated as previously described [45]. Briefly, the mice were anesthetized and decapitated, then the meninges and large vessels were discarded. The hippocampus was dissected and homogenized in a capillary buffer, which was thoroughly mixed with 26% dextran (MW, 70 kDa) so that the final concentration of dextran was slightly more than 13.5%. The tissue homogenate was centrifuged at 5400*g* for 30 min at 4 °C. The microvessels separated from the hippocampus were pelleted at the bottom of the tube.

### RNA isolation and quantitative polymerase chain reaction (qPCR) analysis for enriched microvessels

Total RNA was extracted from pelleted microvessels using TRIzol reagent (TaKaRa) and reversely transcribed to cDNA using the First-Strand cDNA Synthesis kit (Applied Biological Materials). Real-time PCR was performed using SYBR Green PCR Master Mix (Roche) along with cDNA and designed primers on a LightCycler 480 system (Roche). The mRNA expression of genes closely related to the BBB structure and integrity was determined. Relative gene expression was calculated by normalization of the cycling threshold (CT) values against the reference housekeeping gene GAPDH using the 2^−ΔΔCT^ method. The forward and reverse primers used are shown in Additional file 1: Table S2.

### Immunofluorescent assay for isolated microvessels

Freshly isolated hippocampal microvessels were fixed with 2% paraformaldehyde (PFA) for 1 h and then incubated overnight at 4 °C with primary antibodies against Occludin (27260-1-AP, Proteintech) and ZO-1 (21773-1-AP, Proteintech), which were all diluted (1:100) in phosphate-buffered saline (PBS) containing 1% bovine serum albumin and 0.3% Triton X-100. The microvessels were then washed three times with PBS and incubated at 4 °C for an additional 24 h with Alexa Fluor 488-conjugated donkey anti-rabbit secondary antibodies (1:1000, A-21206, Life technologies). After thorough washes, the vessels were mounted and fluorescence images were taken using a Leica DMI 8 microscope. To quantify the target protein signals, the immunofluorescence intensity (IF intensity) was calculated as follows: IF intensity (arbitrary units, a.u.) = integrated density/total fluorescence area. Quantitative analysis was performed with ImageJ software (National Institute of Mental Health).

### Western blot analysis

Hippocampal microvessels were isolated and immediately frozen in liquid nitrogen and stored at − 80 °C. Samples were homogenized in RIPA buffer (FD008, Fd bioscience) supplemented with phosphatase inhibitors (FD1002, Fd bioscience) and protease inhibitors (FD1001, Fd bioscience) on ice for 30 min and centrifuged at 12,000 rpm for 5 min at 4 °C. The supernatant was collected and analyzed with a BCA™ protein assay kit (P0011, Beyotime). The proteins were separated with 12% SDS-PAGE gels and transferred to PVDF membranes (IPVH00010, Millipore). The membranes were blocked in 5% fat-free milk for 2 h at room temperature, and then incubated with the primary antibodies, including anti-ZO-1 antibody (1:5000, 21773-1-AP, Proteintech), ZO-2 antibody (1:1000, 2847, Cell Signaling), anti-Occludin antibody (1:5000, 27260-1-AP, Proteintech), anti-Claudin-1 antibody (1:1000, ab15098, Abcam), anti-Claudin-3 antibody (1:1000, ab15102, Abcam), anti-Claudin-5 antibody (1:1000, ab15106, Abcam) and anti-GAPDH antibody (1:5000, 10494-1-AP, Proteintech) at 4 °C overnight. Goat anti-rabbit HRP-conjugated secondary antibody (1:5000, HS101-01, Transgen) were incubated for 2 h at room temperature. Then, the membranes were visualized by an ECL chemiluminescent detection kit (E411-04, Vazyme) with ChemiDoc MP Imaging System (Bio-Rad). Protein expression was then quantified using ImageJ software (National Institute of Mental Health). The relative expression of the proteins was normalized to that of GAPDH.

### Immunohistochemistry

Mice were euthanized with an overdose of sodium pentobarbital (25 mg/kg, IP) and perfused transcardially with cold PBS until completely exsanguinated followed by 4% PFA for tissue fixation. Brains were removed and submerged in 4% PFA for 24 h at 4 °C, and then dehydrated in 30% sucrose for 24 h at room temperature. For immunostaining of Iba1, coronal brain sections of 40 µm thickness were prepared and incubated with Iba1 antibody (1:500, ab5076, Abcam), the marker of microglia. After washing with PBS, the brain sections were incubated with donkey anti-goat Alexa Fluor 594 conjugated secondary antibody (1:1000, A-11058, Life technologies). Nuclei were counterstained with 4ʹ,6-diamidino-2-phenylindole (DAPI). Meanwhile, GFP^+^ BM-derived cells were identified in brain sections by their autofluorescence in the 488 nm channel. Images were captured using a Zeiss LSM 780 confocal microscope (Carl Zeiss). The number of GFP^+^ cells was counted on at least three brain sections in the hippocampus region per mouse. For quantification of the GFP^+^ recruited cells, data were collected from three serial coronal sections (50 µm) along the anterior–posterior axes of the hippocampus (− 1.82 mm to − 2.8 mm from the bregma) of each mouse. Hippocampus were identified with reference markers in accordance with Paxinos and Franklin’s The Mouse Brain in Stereotaxic Coordinates. The number of GFP^+^ cells was manually counted in a blinded manner from three brain sections per mouse on a 100× field (i.e., 10× objective lens and 10× ocular; 0.81 mm^2^ per field). Data from the three sections were averaged to obtain a single data point for each individual mouse, and the output results were calculated to represent the average number of GFP^+^ cells per area (mm^2^).

### Measurement of BBB permeability with Evans blue

Evans blue perfusion was performed as previously described [46]. Briefly, anesthetized mice were perfused with 50 ml of PBS (pH 7.2) followed by 50 ml of 4% PFA containing 1% Evans blue (Sigma). The dissected brains were post-fixed for 4 h in 4% PFA at 4 °C, and then incubated in 30% sucrose for additional 24 h at 4 °C. Brains were sectioned with a cryotome in 30-μm-thick coronal slices and visualized using a fluorescent microscope (Leica DMI 8) by excitation with 543-nm laser beams (green zone) and visualized as red fluorescence. For the positive control of a “leaky” BBB, mice were intraperitoneally injected with 3 mg/kg of lipopolysaccharide (LPS) to induce the BBB disruption. And then, Evans blue perfusion was performed at 24 h post-injection [47].

### Statistical analysis

All experimental data were presented as the mean ± SEM unless otherwise stated. Normality of the data was tested using Shapiro–Wilk test. The homogeneity of variance was inspected by Levene’s *F* test. For normally distributed data, an unpaired two-sided Student’s *t* test was used to analyze the significance between two experimental groups, while one-way analysis of variance (ANOVA) followed by Turkey’s post hoc test was used for multiple comparisons. When non-normal distribution was detected, either Mann–Whitney test or Kruskal–Wallis one-way ANOVA followed by Dunn’s post hoc test was performed for comparisons between groups where appropriate. GraphPad Prism 8 (GraphPad Software Inc.) and IBM SPSS 23 software (IBM Corp.) were used to process the data, and *P* values < 0.05 were considered statistically significant. To obtain unbiased data, all experiments were carried out by two scientists in a blind manner. Details about statistical analyses for individual experiments are presented in the figure legends.

## Results

### GFP^+^ BM-chimeras were successfully established

**Fig. 1:**
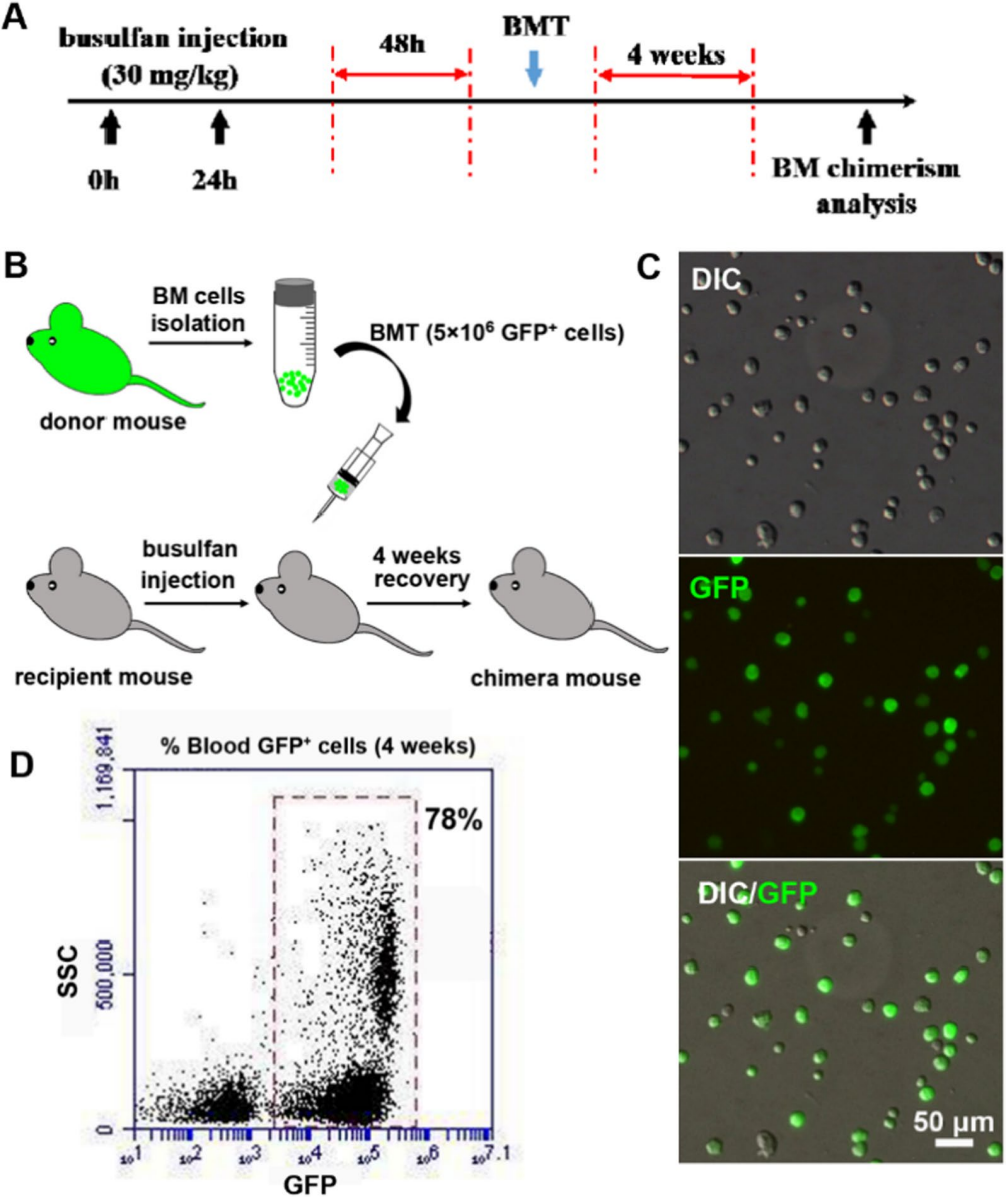
High levels of chimerism (> 78%) were observed 4 weeks post-bone marrow transplantation (BMT). Male C57BL/6J mice were administered with busulfan (30 mg/kg) for 2 consecutive days, and then 5 × 10^6^ GFP^+^ BM cells were injected into the tail vein of recipient mice. The peripheral blood was collected 4 weeks post BMT and GFP^+^ cells were quantified. **A** Schematic drawing of the experiment schedule. **B** BMT process. **C** Representative picture of peripheral blood cytospin slides (*n* = 4 mice from the control group). **D** Representative flow cytometric data of GFP/side scatter (SSC) of peripheral blood (*n* = 4 mice from the control group). Scale bars, 50 μm. GFP, green fluorescent protein. DIC, differential interference contrast

To explore whether BM-derived cells could migrate into the brain from peripheral blood, GFP^+^ BM-chimeras were established using GFP^+^ cells as the indicator of peripheral-derived cells. Male C57/BL6J mice were treated with busulfan for myeloablation and then GFP transgenic mice-derived BM was transplanted (Fig. 1A, B). After 4 weeks, peripheral blood samples were collected and the percentage of GFP^+^ cells was determined. FACS and cytospin slides analysis showed that chimerism levels were > 78% (Fig. 1C, D), which was almost consistent with previously reported data [34].

### CPS induced BM-derived monocyte migration into the hippocampus and differentiation into microglia-like cells

**Fig. 2:**
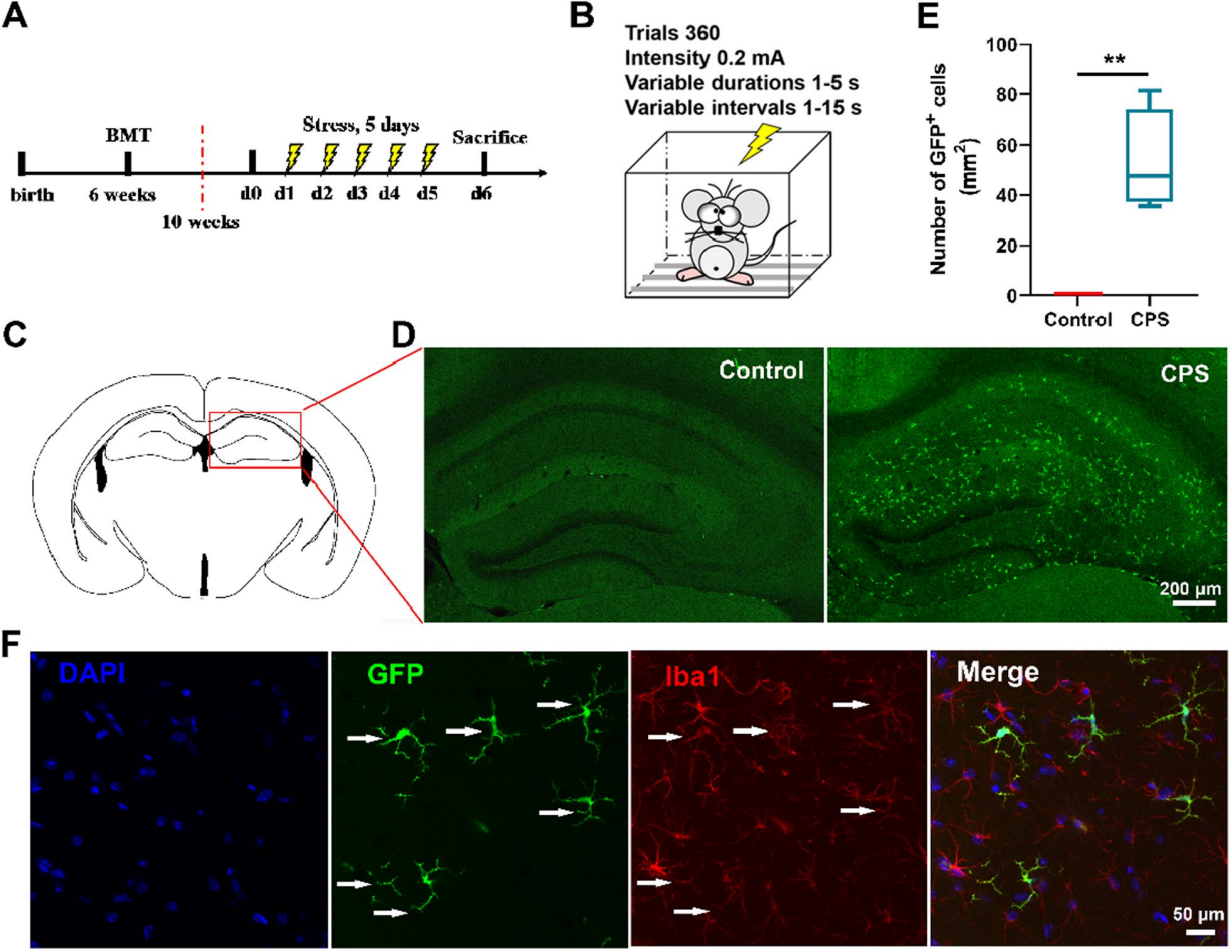
CPS induced BM-derived monocyte migration into the hippocampus and differentiation into microglia-like cells. **A** Schematic of the experimental schedule. Mice received BMT treatment at 6 weeks old, and after 4 weeks, they were exposed to CPS for 5 consecutive days. **B** Schematic representation of CPS treatment (360 trials within 60 min, intensity of 0.2 mA, variable duration of 1–5 s, and variable intervals of 1–15 s). **C** Areas in the red rectangle show the region of the hippocampus in which the representative images were taken. **D** Representative images of GFP^+^ cells (green) in the hippocampus. Scale bars, 200 μm. **E** Quantification of GFP^+^ cells recruited to the hippocampus (*n* = 5 mice/group, Mann–Whitney *U* test, *P* = 0.008). Graphical data are represented as box and whisker plots. The box shows the lower, median and upper quartiles, and the whisker shows the minimum and maximum values. **F** GFP^+^ cells (green) overlapped with Iba1 (red) (*n* = 3 mice/group). Scale bars, 50 μm. **P < 0.01

BM-derived cells have been reported to infiltrate into the brain and interact with resident microglia or differentiate into microglia-like cells to ultimately influence brain function in some neurodegenerative disorders, including Alzheimer’s disease, multiple sclerosis, and amyotrophic lateral sclerosis [48–52]. To further examine whether BM-derived microglia were involved in psychological stress-induced mood disorders, GFP^+^ BM chimeric mice were exposed to CPS treatment (Fig. 2A, B). The results demonstrated that GFP^+^ cells were accumulated in the hippocampus, suggesting that BM-derived cells migrated from peripheral blood into the brain after exposure to CPS (Fig. 2C–E, *P* < 0.001). Moreover, these brain-infiltrating GFP^+^ cells presented a ramified morphology and were positively stained with Iba1, a marker for microglia, indicating that the migrating cells differentiated into microglia-like cells in the hippocampus (Fig. 2F).

### CPS does not affect the integrity of the BBB

**Fig. 3:**
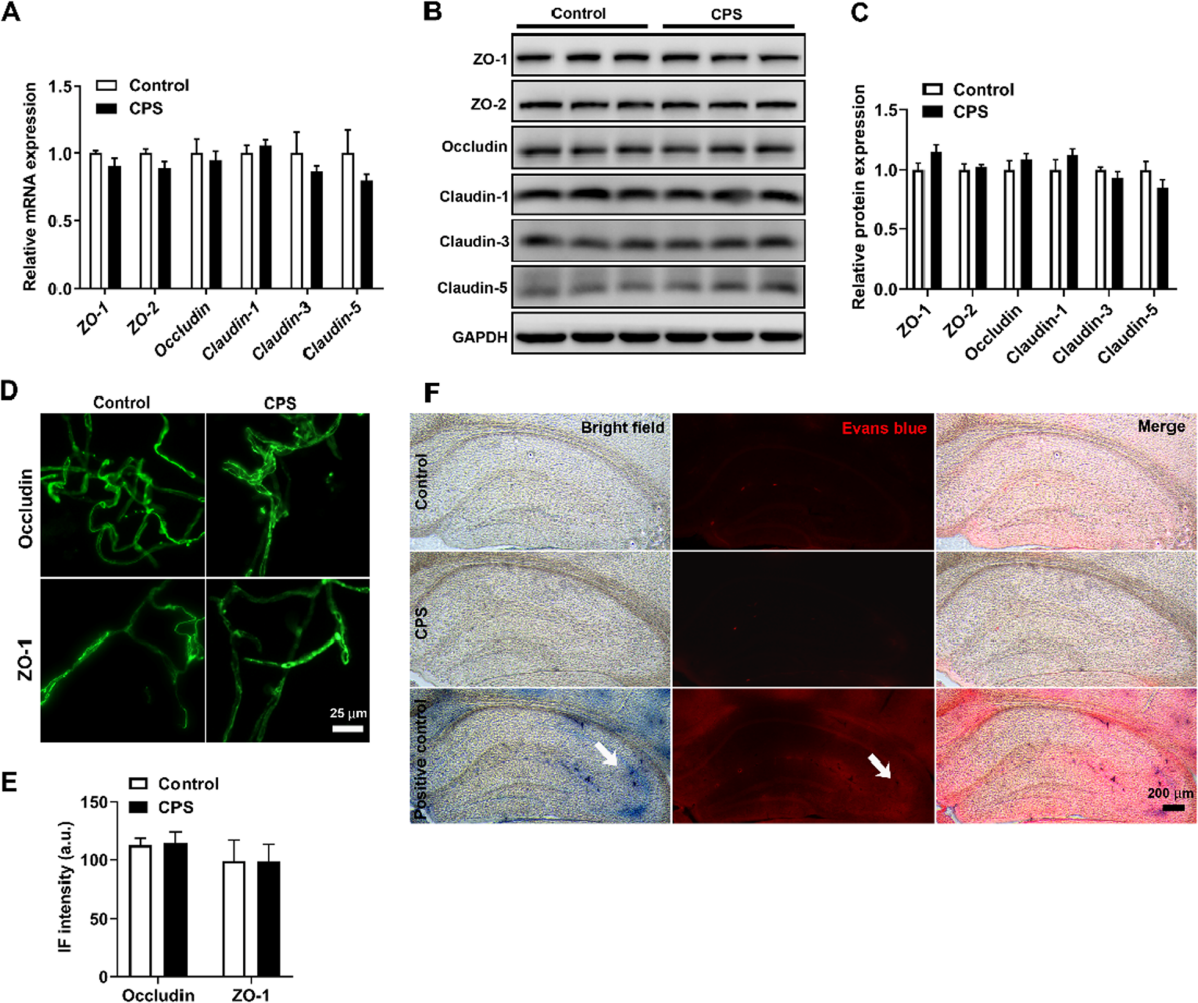
CPS does not affect the integrity of the BBB. **A** qPCR analysis showed no difference in mRNA expression of tight-junction proteins between the two groups (*n* = 4 mice/group, unpaired two-sided Student’s *t* test, *P* > 0.05). **B** Representative western blots showed no difference in the expression of tight-junction proteins between the two groups (*n* = 3 mice/group). **C** Quantitative analysis of tight-junction proteins. Data were normalized to GAPDH expression and expressed as fold change (*n* = 3 mice/group, unpaired two-sided Student’s *t* test, *P* > 0.05). **D** Representative immunofluorescence images of freshly isolated hippocampal microvessels showed intact and continuous staining of Occludin and ZO-1, and no difference was observed between the two groups (*n* = 5 mice/group). Scale bars, 25 μm. **E** Quantification of the immunofluorescence intensity of Occludin and ZO-1 (*n* = 5 mice/group, unpaired two-sided Student’s *t* test, *P* > 0.05). a.u., arbitrary units. **F** Representative images from the hippocampus in which no extravasation of Evans blue was detected in the control and CPS-treated groups compared with the positive control group treated with LPS (*n* = 3 mice/group). White arrows in the positive control group indicate the visible extravasation of Evans blue (Evans blue displays blue color or red color under bright-field or laser beams, respectively). Scale bars, 200 μm. Error bars represent SEM

The BBB, a unique structure of the central nervous system (CNS), is composed of continuous non-fenestrated microvessels. This property allows the BBB to strictly regulate the movement of cells and molecules between the peripheral blood and the CNS [53]. To determine whether central migration of peripheral BM-derived cells depends on the disruption of BBB, BBB permeability under CPS treatment was evaluated. qPCR, western blot and immunofluorescence analysis of tight-junction proteins in isolated hippocampal microvessels were performed, and Evans blue extravasation was visualized using a fluorescent microscope. Compared with the control groups, no difference was found in the mRNA (Fig. 3A) and protein (Fig. 3B, C) expression of *ZO-1*, *ZO-2*, *Occludin*, *Claudin-1*, *Claudin-3*, and *Claudin-5*, all these tight-junction protein genes are key molecules in the formation of structural backbones of the BBB. Immunofluorescence staining also confirmed that there were no differences in the expression of Occludin and ZO-1 in the isolated hippocampal microvessels in the CPS group compared with the control group (Fig. 3D, E). Moreover, compared with the positive control group treated with LPS, CPS did not influence the BBB integrity, as no extravasation of Evans blue was detected in the hippocampus (Fig. 3F). Taken together, these data reveal that CPS has no destructive effect on the integrity of BBB, indicating that central migration of peripheral BM-derived cells was not associated with increased BBB permeability.

### RS102895 alleviated CPS-induced depressive-like behaviors by inhibiting the infiltration of BM-derived monocytes into the hippocampus

**Fig. 4:**
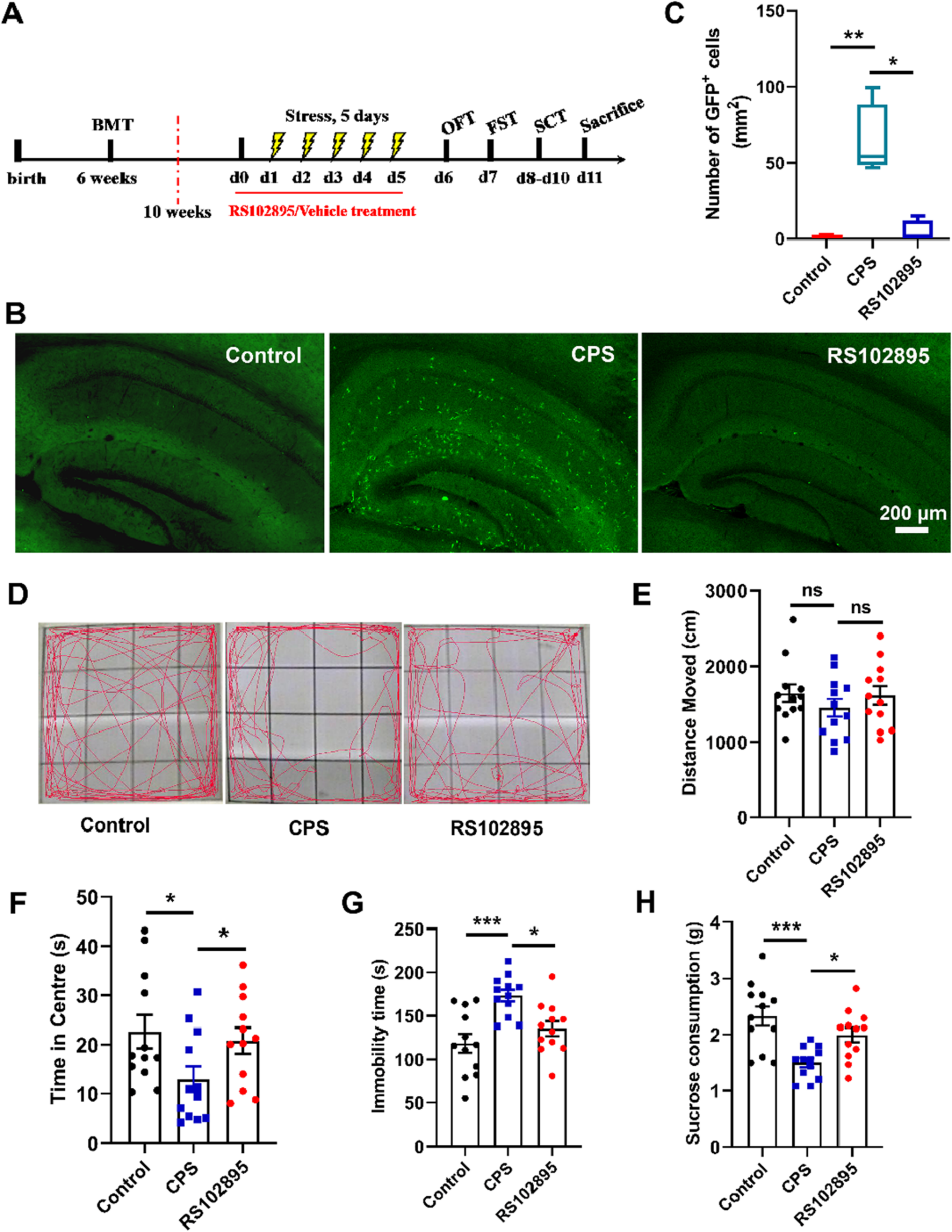
RS102895 alleviated CPS-induced depressive-like behaviors by inhibiting the infiltration of BM-derived monocytes into the hippocampus. **A** Schematic of the experimental schedule. After successful BMT, mice received the treatment of RS102895 or vehicle from 1 day before CPS induction until the end of CPS exposure. Finally, the mice underwent behavioral tests. **B** Representative image of BM-derived GFP^+^ cells (green) in the hippocampus (*n* = 4 mice/group). RS102895 suppressed the infiltration of BM-derived GFP^+^ cells into the hippocampus. Scale bars, 200 μm. **C** Quantification of GFP^+^ cells recruited to the hippocampus (*n* = 4 mice/group, Kruskal–Wallis one-way ANOVA, *P* = 0.020). Graphical data are represented as box and whisker plots. The box shows the lower, median and upper quartiles, and the whisker shows the minimum and maximum values. **D** Representative trajectories of indicated mice in OFT (*n* = 12 mice/group). **E** Total distance traveled within each 5-min period in indicated groups in OFT (*n* = 12 mice/group, one-way ANOVA, *F*_(2, 33)_ = 0.762, *P* = 0.475). **F** Time spent in the central zone in indicated groups in OFT (*n* = 12 mice/group, one-way ANOVA, *F*_(2, 33)_ = 4.960, *P* = 0.013). **G** Immobility time of mice in indicated groups in FST (*n* = 12 mice/group, one-way ANOVA, *F*_(2, 33)_ = 10.093, *P* < 0.001). **H** Sucrose consumption of mice in indicated groups in SCT (*n* = 12 mice/group, one-way ANOVA, *F*_(2, 33)_ = 9.933, *P* < 0.001). *P < 0.05, ***P < 0.001. ns, not significance. Error bars represent SEM

To further explore the role of BM-derived cells in CPS-induced depressive-like behaviors, RS102895, a CCR2 antagonist, was applied to mice. RS102895 was shown to specifically bind to the β-subunit of the CCR2 receptor with relatively high affinity, resulting in potent inhibition of CCR2 signaling [33]. Previous studies have shown that RS102895 at a dose of 5 mg/kg could reduce monocyte recruitment to the brain in mice exposed to chronic partial sciatic nerve ligation and antibiotic treatment [34, 44]. Therefore, after successful BMT, mice in this study were orally administered with RS102895 or equivalent vehicle every 12 h starting from 1 day before CPS induction for 6 consecutive days. Afterwards, the behavioral tests were performed in the sequence of OFT, FST, and SCT (Fig. 4A). The results demonstrated that RS102895 significantly suppressed the recruitment of BM-derived GFP^+^ cells into the hippocampus (Fig. 4B, C, *P* < 0.001). As expected, CPS induced depressive-like behaviors, including decreased time in the central zone in the OFT (Fig. 4D, F, *P* < 0.05), increased immobility time in the FST (Fig. 4G, *P* < 0.001), and reduced sucrose consumption (Fig. 4H, *P* < 0.001), which were alleviated by administration of RS102895. In addition, no difference was detected in the total travel distance in the OFT test among the 3 groups (Fig. 4E). To further measure anhedonia more directly in the mice subjects, SPT was also conducted. The results showed that the sucrose preference was significantly decreased in CPS-treated mice compared to unstressed control mice. With RS102895 treatment, mice exhibited significantly higher sucrose preference than CPS-treated mice, confirming the antidepressant effect induced by RS102895 treatment (Additional file 1: Fig. S1). Collectively, these results suggested that RS102895 alleviated CPS-induced depressive-like behaviors by inhibiting the infiltration of BM-derived monocytes into the hippocampus.

## Discussion

The present study demonstrates that CPS induces the migration of peripheral BM-derived monocytes into the hippocampus. These monocytes differentiate into microglia-like cells after infiltration into the brain. While inhibiting the infiltration of BM-derived monocytes into the hippocampus, depressive-like behavior changes were alleviated. More importantly, central migration of peripheral BM-derived cells was not associated with BBB disruption, as the expression of tight-junction proteins of the brain blood vessels and their permeability were not changed under CPS. These findings indicate that monocyte recruitment to the hippocampus in response to psychological stress may represent a novel cellular mechanism that contributes to the development of depression, adding to our understanding of the mechanism by which CPS contributes to mood disorders.

Several lines of evidence indicate that BM-derived cells are involved in psychiatric disorders induced by chronic stress. To track the migration of BM-derived cells in our CPS mouse model, GFP^+^ BM-chimeras were established, in which endogenous BM cells were replaced with BM cells ubiquitously expressing GFP from transgenic C57BL/6 mice donors. This procedure allowed localization and observation of the morphology of GFP^+^ BM-derived cells within the brain using immunohistochemistry. For BM transplantation experiments, BM cells of recipient mice were eliminated by intraperitoneal injection with busulfan. Compared with body irradiation-based myeloablative regimen, no specialized facility and equipment are required by this approach. In addition, potentially lethal damage to other tissues is reduced. After 4 weeks of BMT, chimerism levels > 78% were typically established in the peripheral blood, which was almost consistent with our previous result [34]. The BMT model is an invaluable tool for studying bidirectional communication between the peripheral immune system and the CNS.

Microglia, the resident innate immune cells in the brain, play a crucial role in the neuroinflammatory response and pathogenesis of neurodegenerative diseases such as Parkinson’s disease and Alzheimer’s disease, as well as various psychiatric disorders, including depression and anxiety [54, 55]. When microglia are activated, they exert a similar immune function as peripheral macrophages, producing inflammatory cytokines, chemokines, and prostaglandins [56]. Besides, changes in the morphology and function of microglia, as well as an increase in the number of microglia are also observed in CNS diseases. One hypothesis is that peripheral monocytes or other BM-derived progenitors contribute to the microglia pool [57]. Interestingly, in our CPS mouse model, it was observed that BM-derived cells infiltrated into the hippocampus and differentiated into microglia-like cells, as GFP^+^ cells overlapped with microglial marker Iba1. However, whether the recruitment of monocytes or other BM-derived progenitors contributes to the CNS resident microglia pool remains a topic of considerable debate. Studies in stress and neurological disease models, where infiltrating GFP^+^ macrophages were reduced in the brain or spinal cord with time suggested that BM-derived monocytes did not contribute to the long-term microglial pool [58–61]. Due to the limitations in time, the present study does not provide any data on long-term observation of BM-derived monocytes in CPS-treated mice, which warrants further exploration.

Recently, Picard et al. revealed that glucocorticoid receptors (GR) expressed by microglia are important for the alteration of microglial function. Under chronic stress conditions, GR depleted-microglia showed reduced expression levels of pro-inflammatory genes and increased neuroprotective genes compared to WT-microglia [62]. Activated microglia then promote HPA responses, releasing more glucocorticoids into circulation, which reaches various organs, including the brain and BM. Myeloid cells in the BM proliferate after receiving the signal and migrate into the brain, forming positive feedback and exacerbating inflammation. Therefore, the mechanisms responsible for the migration and functions of microglia-like infiltrated cells in terms of neurogenesis and mood disorder need further investigation.

At present, it is possible to define three distinct routes for leukocytes entry into the brain: from blood to cerebrospinal fluid (CSF) across the choroid plexus; from blood to the subarachnoid space through meningeal vessels; and from blood to parenchymal perivascular spaces directly crossing the BBB [24]. Our previous study demonstrated that antibiotic-induced microbiome depletion disrupts the BBB and facilitates infiltration of BM-derived monocyte into the brain [34]. However, in the current study, the central migration of peripheral BM-derived cells was not associated with BBB disruption, because the expression of tight-junction proteins of the brain blood vessels and their permeability did not change under CPS. Moreover, Taghadosi et al. recently reported that rats exposed to chronic electric foot shock stress exhibited increased hippocampal BBB permeability [63]. The discrepancy may be attributed to the different mouse models and analytical methods. In the study conducted by Taghadosi et al., rats were exposed to much stronger foot shock tress (1 mA, 1 Hz, for 10-s duration every 60 s (1 h/day) for 10 consecutive days) than we used. In addition, to assess whether CPS influences BBB permeability, qPCR and immunofluorescence analysis of tight-junction proteins in isolated hippocampal microvessels were performed, and Evans blue extravasation was visualized using a fluorescent microscope in the present study. However, Taghadosi and colleagues only performed the Evans blue extravasation assay, which was determined by absorbance of hippocampus supernatants for Evans blue and macroscopic evaluation. Overall, these findings suggest that the central migration of peripheral BM-derived cells could be mediated by the first two routes independent of the third one in CPS-treated mice.

CPS-induced depressive-like behaviors, including decreased time in the central zone in OFT, increased immobility time in FST, and reduced sucrose intake, were associated with BM-derived monocytes infiltration into the hippocampus. To further explore the role of BM-derived cells in these symptoms, RS102895, a CCR2 antagonist, was applied to mice to inhibit BM-derived monocyte infiltration. The oral gavage of RS102895 at a dose of 5 mg/kg twice daily for 15 consecutive days demonstrated no obvious influence on the behavioral phenotypes of male C57BL/6J mice on the background of BMT (Additional file 1: Fig. S2). However, when concurrently treated with CPS, RS102895 alleviated CPS-induced depressive-like behaviors by inhibiting the infiltration of BM-derived monocytes into the hippocampus. Similar to our findings, Sawada et al. demonstrated that suppression of BM-derived microglia in the amygdala improved anxiety-like behavior induced by chronic partial sciatic nerve ligation in mice [44]. Wohleb et al. found that stress-induced recruitment of BM-derived monocytes to the brain promoted anxiety-like behavior [35]. Together, these findings indicate that monocyte recruitment to the hippocampus in response to psychological stress may represent a novel cellular mechanism that contributes to the development of depression, adding to our understanding of the mechanism by which CPS contributes to mood disorders.

## Supplementary Information

**Additional file 1. Table S1.** Mice numbers (N) for each experiment in this study. **Table S2.** Primer sequence used for qPCR analysis. **Fig. S1.** The results of sucrose preference in the SPT. The sucrose preference in the SPT was significantly decreased in CPS-treated mice compared to unstressed control mice. With RS102895 treatment, mice exhibited significantly higher sucrose preference than CPS-treated mice, confirming the antidepressant effect induced by the CCR2 antagonist (n = 14 mice/group, one-way ANOVA, F (2, 39) = 5.457, P = 0.008). *P < 0.05, **P < 0.01. Error bars represent SEM. **Fig. S2.** The effects of RS102895 treatment on depressive-like behaviors. **A.** Representative trajectories of indicated mice in OFT (n=10 mice/group). **B.** Total distance traveled within each 5-min period in indicated groups in OFT (n=10 mice/group, unpaired two-sided Student’s t test, P = 0.930). **C.** Time spent in the central zone in indicated groups in OFT (n=10 mice/group, unpaired two-sided Student’s t test, P = 0.567). **D.** Immobility time of mice in indicated groups in FST (n=10 mice/group, unpaired two-sided Student’s t test, P = 0.713). **E.** Sucrose consumption of mice in indicated groups in SCT (n=10 mice/group, unpaired two-sided Student’s t test, P = 0.659). ns, not significant. Error bars represent SEM.

## Data Availability

The data in this study are available from the corresponding author upon reasonable request.

## Abbreviations

BM: Bone marrow
CPS: Chronic psychological stress
BBB: Blood–brain barrier
GFP: Green fluorescent protein
CCR2: C–C chemokine receptor type 2
PFC: Prefrontal cortex
HPA: Hypothalamic pituitary adrenal axis
SNS: Sympathetic nervous system
OFT: Open field test
FST: Forced swim test
SCT: Sucrose consumption test
LPS: Lipopolysaccharide
