# Supplementary material for: Psychological stress induces depressive-like behavior associated with bone marrow-derived monocyte infiltration into the hippocampus independent of blood–brain barrier disruption
Source: J Neuroinflammation. 2022 Aug 24;19:208. doi: 10.1186/s12974-022-02569-w (PMC9400267; doi:10.1186/s12974-022-02569-w)
Supplement: Supplementary file 1 — Additional file 1. Table S1. Mice numbers (N) for each experiment in this study. Table S2. Primer sequence used for qPCR analysis. Fig. S1. The results of sucrose preference in the SPT. The sucrose preference in the SPT was significantly decreased in CPS-treated mice compared to unstressed control mice. With RS102895 treatment, mice exhibited significantly higher sucrose preference than CPS-treated mice, confirming the antidepressant effect induced by the CCR2 antagonist (n = 14 mice/group, one-way ANOVA, F (2, 39) = 5.457, P = 0.008). *P < 0.05, **P < 0.01. Error bars represent SEM. Fig. S2. The effects of RS102895 treatment on depressive-like behaviors. A. Representative trajectories of indicated mice in OFT (n=10 mice/group). B. Total distance traveled within each 5-min period in indicated groups in OFT (n=10 mice/group, unpaired two-sided Student’s t test, P = 0.930). C. Time spent in the central zone in indicated groups in OFT (n=10 mice/group, unpaired two-sided Student’s t test, P = 0.567). D. Immobility time of mice in indicated groups in FST (n=10 mice/group, unpaired two-sided Student’s t test, P = 0.713). E. Sucrose consumption of mice in indicated groups in SCT (n=10 mice/group, unpaired two-sided Student’s t test, P = 0.659). ns, not significant. Error bars represent SEM. [file 12974_2022_2569_MOESM1_ESM.docx]

**Additional Information for**

**Psychological stress induces depressive-like behavior associated with bone marrow-derived monocyte infiltration into the hippocampus independent of blood-brain barrier disruption**

Huiling Hu^1,2,*^, Xue Yang^3,*^, Yuqing He^4^, Chaohui Duan^1,2,#^, Nannan Sun^5,#^

^*^These authors contributed equally to this work.

^#^ To whom correspondence should be:

Chaohui Duan, E-mail: duanchh@mail.sysu.edu.cn

Nannan Sun, E-mail: sun_nann@163.com

This file includes:

Extended Materials and Method

Fig. S1 to S2.

Table S1 to Table S2.

**Extended Materials and Method**

**Sucrose Preference Test (SPT)**

The SPT was another behavioral test adopted to signal stress-induced anhedonic symptom. Mice were singly housed and allowed to habituate to 1% sucrose solution (w/v) for 3 days with free access to two identical 50 mL bottles containing pure water and sucrose solution respectively. The position of each bottle was rotated every 24 hours to prevent position preference in the subjects. After adaptation, the mice were deprived of both food and water for 24 h. Then, a 2-hour preference test was conducted in which pure water and 1% sucrose solution (w/v) were simultaneously delivered to the mice again. The bottle positions were switched every 1 hour to avoid a side bias. The sucrose preference (%) was determined by weighing the drinking bottles at the start and end of the testing period, and presented as the ratio of sucrose solution consumption to the total fluid intake.





Fig. S1. The results of sucrose preference in the SPT. The sucrose preference in the SPT was significantly decreased in CPS-treated mice compared to unstressed control mice. With RS102895 treatment, mice exhibited significantly higher sucrose preference than CPS-treated mice, confirming the antidepressant effect induced by the CCR2 antagonist (n = 14 mice/group, one-way ANOVA, F _(2, 39)_ = 5.457, P = 0.008). *P < 0.05, **P < 0.01. Error bars represent SEM.


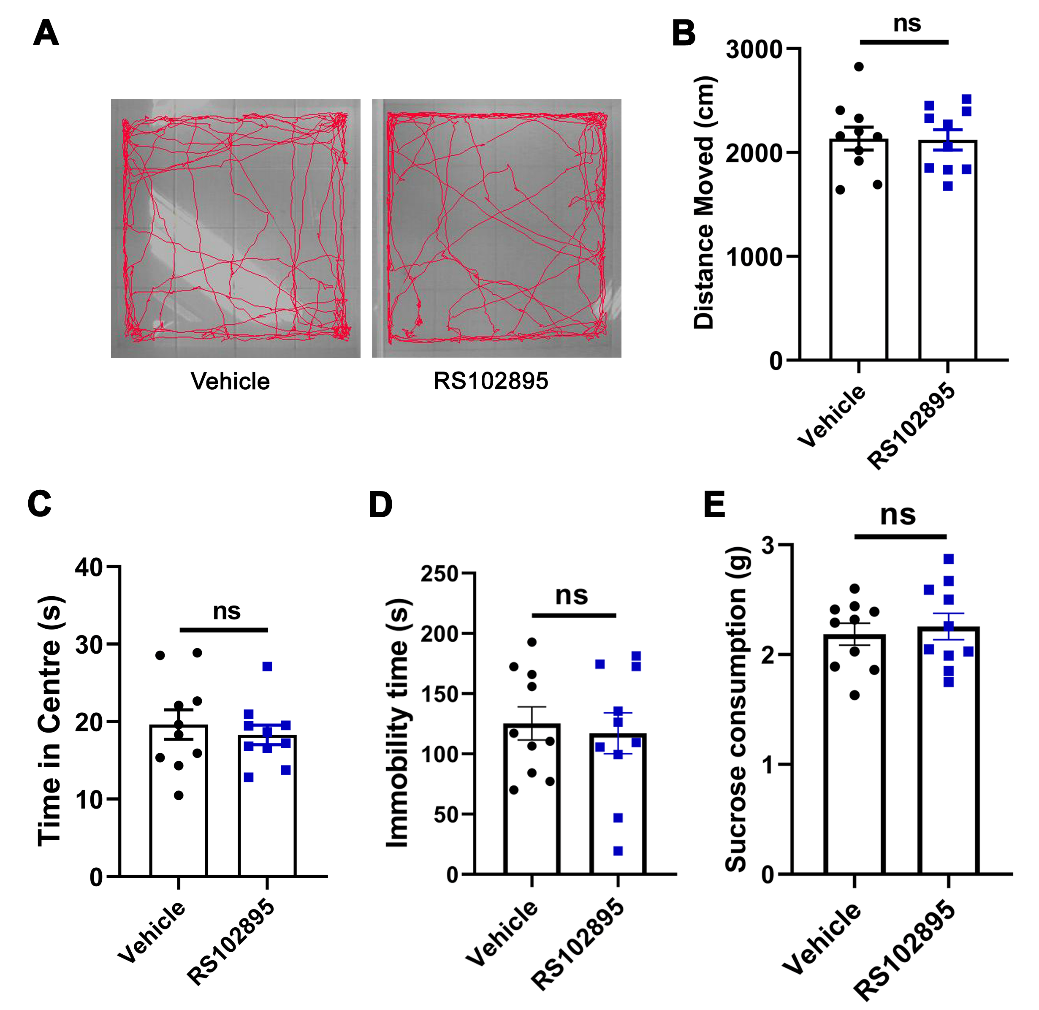


Fig. S2. The effects of RS102895 treatment on depressive-like behaviors. A. Representative trajectories of indicated mice in OFT (n=10 mice/group). B. Total distance traveled within each 5-min period in indicated groups in OFT (n=10 mice/group, unpaired two-sided Student’s t test, P = 0.930). C. Time spent in the central zone in indicated groups in OFT (n=10 mice/group, unpaired two-sided Student’s t test, P = 0.567). D. Immobility time of mice in indicated groups in FST (n=10 mice/group, unpaired two-sided Student’s t test, P = 0.713). E. Sucrose consumption of mice in indicated groups in SCT (n=10 mice/group, unpaired two-sided Student’s t test, P = 0.659). ns, not significant. Error bars represent SEM.

Table S1. Mice numbers (N) for each experiment in this study.

| Cohort 1 (BMT and BMT+CPS), N=40 | | | | | | |
| --- | --- | --- | --- | --- | --- | --- |
| Figure 1 | | | | | | |
|  | BMT mouse | | | | Total | Age at Assessment |
| N (Fig. 1C-D) | 4 (overlapped with Fig. 2-3) | | | | 4 | 11 weeks |
| Figure 2 | | | | | | |
|  | Control | | CPS | | Total | Age at Assessment |
| N (Fig. 2C-E) | 5 | | 5 | | 16 | 11 weeks |
| N (Fig. 2F) | 3 | | 3 | |  |  |
| Figure 3 | | | | | | |
|  | Control | | CPS | | Total | Age at Assessment |
| N (Fig. 3A) | 4 | | 4 | | 24 | 11 weeks |
| N (Fig. 3B-C) | 3 (overlapped with Fig. 3A) | | 3 (overlapped with Fig. 3A) | |  |  |
| N (Fig. 3D-E) | 5 | | 5 | |  |  |
| N (Fig. 3F) | 3 | | 3 | |  |  |
| Cohort 2 (BMT and BMT+CPS and BMT+CPS+RS102895), N=48 | | | | | | |
| Figure 4 | | | | | | |
|  | Control | CPS | | RS102895 | Total | Age at Assessment |
| N (Fig. 4B-C) | 4 | 4 | | 4 | 48 | 12 weeks |
| N (Fig. 4D-F) | 12 | 12 | | 12 |  |  |
| N (Fig. 4G) |  |  |  |  |  |  |
| N (Fig. 4H) |  |  |  |  |  |  |
| Cohort 3 (BMT and BMT+CPS and BMT+CPS+RS102895), N=28 | | | | | | |
| Figure S1 | | | | | | |
|  | Vehicle | | RS102895 | | Total | Age at Assessment |
| N (Fig. S1) | 14 | | 14 | | 28 | 12 weeks |
| Cohort 4 (BMT and BMT+RS102895), N=20 | | | | | | |
| Figure S2 | | | | | | |
|  | Vehicle | | RS102895 | | Total | Age at Assessment |
| N (Fig. S2. A-C) | 10 | | 10 | | 20 | 12 weeks |
| N (Fig. S2. D) |  |  |  |  |  |  |
| N (Fig. S2. E) |  |  |  |  |  |  |

**Table S2. Primer sequence used for qPCR analysis**

| **Gene** | **Primer sequence (5′–3′)** | |
| --- | --- | --- |
| *Claudin-1* | Forward: | ACTCCTTGCTGAATCTGAACAGT |
|  | Reverse: | GGACACAAAGATTGCGATCAG |
| *Claudin-3* | Forward: | TGGGAGGGCCTGTGGAT |
|  | Reverse: | CGTACATTTTGCACTGCATCTGA |
| *Claudin-5* | Forward: | CTGCCTTCCTGGACCACAA |
|  | Reverse: | TCCACAGCCCCTTCCAAGT |
| *Occludin* | Forward: | TCAAACCGAATCATTATGCACCA |
|  | Reverse: | AGATGGCAATGCACATCACAA |
| *ZO-1* | Forward: | GAGCTACGCTTGCCACACTGT |
|  | Reverse: | TCGGATCTCCAGGAAGACACTT |
| *ZO-2* | Forward: | CTTCTCACATTCAAAGTGGCTTC |
|  | Reverse: | CTACCTGCTAGAAATCCCCAAA |
| *GAPDH* | Forward: | TGTGTCCGTCGTGGATCTGA |
|  | Reverse: | CCTGCTTCACCACCTTCTTGA |
